# Changing Disease Course of Crimean-Congo Hemorrhagic Fever in Children, Turkey

**DOI:** 10.3201/eid2902.220976

**Published:** 2023-02

**Authors:** Pembe Derin Oygar, Sibel Laçinel Gürlevik, Erdal Sağ, Sare İlbay, Tekin Aksu, Osman Oğuz Demir, Yasemin Coşgun, Selin Aytaç Eyüpoğlu, Jale Karakaya, Şule Ünal Cangül, Ali Bülent Cengiz, Yasemin Özsürekci

**Affiliations:** Hacettepe University, Ankara, Turkey (P.D. Oygar, S.L. Gürlevik, E. Sağ, T. Aksu, O.O. Demir, S.A. Eyüpoğlu, J. Karakaya, Ş.Ü. Cangül, A.B. Cengiz, Y. Özsürekci);; İhsan Doğramacı Children’s Hospital, Ankara (P.D. Oygar, S.L. Gürlevik, T. Aksu, O.O. Demir, S.A. Eyüpoğlu, Ş.Ü. Cangül, A.B. Cengiz, Y. Özsürekci);; Ankara Training and Research Hospital, Ankara (E. Sağ);; Hacettepe University Rheumatology Translational Medicine Laboratories, Ankara (S. İlbay);; General Directorate of Public Health, Ministry of Health, Microbiology Reference and Biological Materials Laboratory, Ankara (Y. Coşgun)

**Keywords:** Crimean-Congo hemorrhagic fever, CCHF, viral hemorrhagic fevers, viruses, febrile infections, children, cytokines, chemokines, HLH, hemophagocytic lymphohistiocytosis, MIS-C, multisystem inflammatory syndrome in children, Turkey

## Abstract

Crimean-Congo hemorrhagic fever (CCHF), endemic in certain regions of the world, is listed as a priority disease with pandemic potential. Since CCHF was first identified in Turkey, children have been known to experience milder disease than adults. However, during the COVID-19 pandemic, we observed an unusually severe disease course, including hemophagocytic lymphohistiocytosis (HLH). We examined cytokine/chemokine profiles of 9/12 case-patients compared with healthy controls at 3 time intervals. Interferon pathway–related cytokines/chemokines, including interleukin (IL) 18, macrophage inflammatory protein 3α, and IL-33, were elevated, but tumor necrosis factor-α, IL-6, CXCL8 (formerly IL-8), and cytokines acting through C-C chemokine receptor 2 and CCR5 were lower among case-patients than controls. Interferon pathway activation and cytokines/chemokines acting through CCR2 and CCR5 improved health results among children with severe CCHF. Children can experience severe CCHF, including HLH, and HLH secondary to CCHF can be successfully treated with intravenous immunoglobulin and steroid therapy.

Crimean-Congo hemorrhagic fever (CCHF) is a tickborne viral disease widely distributed throughout the world (*1*). CCHF virus (order *Bunyavirales*, family *Nairoviridae*, genus *Orthonairovirus*) causes disease only in humans, but its natural cycle includes wild mammals, livestock, birds, and ticks. The virus has been detected in >35 tick species; *Hyalomma* genus ticks are recognized vectors, and *H. marginatum* ticks are the most efficient vectors (*2*). Transmission by direct contact with infected human or animal secretions, fluids, or tissues, and nosocomial outbreaks, including transmission related to aerosol generation, have also been reported (*3**–**9*). 

The course of CCHF ranges from asymptomatic to severe (*3*) and even fatal in 3%–40% of cases (*4*). Hemorrhagic complications and hemophagocytic lymphohistiocytosis (HLH) are usually responsible for deaths among adults; CCHF is usually mild in children for reasons that remain unclear. Severe disease and fatalities in adults have been attributed to high viral loads and excessive cytokine release (*10*,*11*). Previous studies have reported varying results in which proinflammatory cytokines are responsible for severe and fatal CCHF. One study (*10*) reported interferon-γ (IFN-γ), interleukin (IL) 10 (IL-10), and tumor necrosis factor-α (TNF-α) to be associated with high viral load and disease severity. Another reported that IL-10, C-X-C motif chemokine ligand 10 (CXCL-10, formerly IFN-γ inducible protein 10) and CC chemokine ligand 2 (CCL2, formerly monocyte chemoattractant protein 1) levels were higher in patients with high viral loads (*12*), but patients with severe disease had higher levels of CXCL10 and CCL2 than did patients with less-severe cases. IL-6 and CXCL-8 (formerly IL-8) were proposed as the main cytokines predicting fatality in 1 study (*13*); another study reported that TNF-α and IL-6 levels were higher in fatal cases (*14*). Another study comparing cytokine levels between adults and children revealed that IL-2, IL-5, IL-9, IL-12p70, and IL-13 were higher in fatal cases among adults. Although some pediatric patients have died, their cytokine profiles did not differ from those for adults or other children on the basis of disease severity (*15*). 

Since 2015, CCHF has been considered one of the emerging infectious diseases most likely to cause major epidemics; at present, it is listed by the World Health Organization as a priority disease with pandemic potential (*16*). During the COVID-19 pandemic, we observed an increased number of pediatric CCHF cases in Turkey associated with unexpectedly severe disease, including certain cases referred to or misdiagnosed as multisystem inflammatory syndrome in children (MIS-C) (*17*). In this study, we aimed to explain the reasons for variations in disease severity among children by determining cytokine/chemokine profiles over time, as well as evaluating clinical and laboratory parameters of patients. 

## Materials and Methods

### Study Design and Patients

We conducted a retrospective study of 12 patients with PCR-confirmed CCHF and 11 healthy volunteers as a control population during April 22, 2020–August 31, 2021, at Hacettepe University İhsan Doğramacı Children’s Hospital in Ankara, Turkey. We obtained demographic information, self-reported times of tick bites and onset of symptoms, and results from physical examinations and diagnostic tests from patient medical records. We performed quantitative reverse transcription PCR (qRT-PCR) targeting CCHF virus for all patients at the Turkey Public Health Reference Laboratory (PHRL). 

All 12 patients seeking treatment were diagnosed with HLH on the basis of criteria from the Histiocyte Society HLH-2004 study (*18*). We tested all CCHF patients for SARS-CoV-2 at admission by using qRT-PCR targeting SARS-CoV-2 with primers supplied by PHRL. The volunteer controls had no history, signs or symptoms, or physical findings of disease and no underlying conditions. Because the study was performed during the pandemic, we also tested controls for SARS-CoV-2 to eliminate the possibility of asymptomatic COVID-19. We obtained informed written consent from guardians of all patients and controls. Our study was approved by the Hacettepe University Ethics Committee (GO21/413). 

### Serum Collection and Laboratory Tests

For each CCHF patient, we measured complete blood count with differentials, C-reactive protein (CRP), erythrocyte sedimentation rate (ESR), aspartate aminotransferase (AST), alanine aminotransferase (ALT), creatinine kinase (CK), lactate dehydrogenase (LDH), ferritin, prothrombin time, activated partial thromboplastin time (aPTT), international normalized ratio (INR), fibrinogen, D-dimer, triglycerides, sodium, potassium, blood urea nitrogen, albumin, and glucose at admission and every 8–12 h until laboratory findings were at safe levels, then daily. At 8–12, 48–60, and 96–120 h after admission, we collected an additional 3 sets of serum samples in addition to the HLH marker samples, centrifuged them at 4,000 rpm for 10 min at room temperature, and stored them at −80°C until used for cytokine/chemokine profiling. We obtained blood samples from controls once and stored them at −80°C until used as group measures for cytokine/chemokine profiling. 

### Cytokine/Chemokine Profiling

We studied serum cytokine/chemokine levels from the serum samples collected 8–12, 48–60, and 96–120 h after admission using cytometric bead-based multiplex assay panels according to manufacturer instructions. Then, we analyzed them using the Agilent Novocyte 3005 flow cytometer (https://www.agilent.com). We used Biolegend LEGENDplex Human Inflammation [13-plex] panel 1 (https://www.biolegend.com) for panel 1 for IL-1β, IFN-α2, IFN-γ, TNF-α, CCL2, IL-6, CXCL8, IL-10, IL-12p70, IL-17A, IL-18, IL-23, and IL-33. We used Biolegend LEGENDplex HU proinflammatory chemokine panel 1 [13-plex] for panel 2 for CCL2, CCL5 (formerly regulated on activation, normal T-cell expressed and secreted [RANTES]), CXCL10, CCL11 (formerly Eotaxin), CCL17 (formerly thymus- and activation-regulated chemokine [TARC]), CCL3 (formerly macrophage inflammatory proteins-1α [MIP-1α]), CCL4 (formerly MIP-1β) CXCL9 (formerly monokine-induced γ interferon [MIG]), CCL20 (formerly MIP-3α), CXCL5 (formerly epithelial neutrophil-activating-78 [ENA-78]), CXCL1 (formerly growth related protein α [GRO-α]), CXCL11 (formerly interferon-inducible T-cell alpha chemoattractant [I-TAC]), and CXCL8. For 9 CCHF patients, we adjusted time intervals for cytokine/chemokine measurements on the basis of first day of self- or parent-reported symptoms, rather than day of hospital admission. We then studied cytokine/chemokine levels at 3 subsequent adjusted time intervals: first (68–72 h after baseline), second (120–132 h), and third (156–180 h). 

### Statistical Analysis

We used IBM SPSS Statistics 26 (https://www.ibm.com) for all statistical analyses. We expressed categorical variables as frequencies and percentages and continuous variables as medians with interquartile ranges (IQRs). To compare cytokine/chemokine levels between patient and control groups, we used the Mann-Whitney U test and set p <0.05 as statistically significant. We performed Friedman 2-way analysis of variance of ranks to determine the changes in cytokine/chemokine and laboratory parameter levels over the 3 time intervals. We calculated the time interval contributing to change using Pearson pairwise comparison with Bonferroni correction. Finally, we determined correlation between laboratory tests and cytokine/chemokine levels with Pearson correlation analysis. We used GraphPad Prism version 9.2.0 (https://www.graphpad.com) for figure configurations. 

## Results

### Demographics and Clinical Findings for Patients and Controls 

We included 23 children, 12 with diagnosed CCHF (3 girls, 9 boys; median age 12.5 [IQR 6–15] years) and 11 controls (6 boys, 5 girls; median age 7 [IQR 2–16] years) in the study. We recorded age, initial symptoms, major pathologic physical examination findings, treatments, and outcomes for all 12 CCHF patients (Table 1). All 12 had a history of tick bites and came from endemic regions; 9 (75%) had close contact with livestock. None had underlying conditions. All patients had fever when initially seeking treatment; the second most common complaint was headache (n = 6, 50%), followed by nausea (n = 4, 33.3%) and malaise (n = 4, 33.3%). Two patients (16.7%) had vomiting and diarrhea. The 2 patients (16.7%) with longest times, 8 and 12 days, from tick bite until hospital admission had myalgia. Median duration of symptoms was 2.5 (IQR 2–5) days; median time between tick bite and baseline was 5.5 (IQR 3–12) days. All patients had conjunctival injection, facial hyperemia, and hepatosplenomegaly at presentation. Seven (58%) patients had petechia at hospital admission, 4 (30%) had findings consistent with central nervous system involvement, 3 (25%) exhibited confusion, and 1 (0.8%) had somnolence; none of the patients had gross bleeding. Median duration of fever after hospital admission was 4 (IQR 3–6) days; median hospital stay was 10 (IQR 5–10) days. 

**Table 1:** Demographic and clinical characteristics, management, and outcomes of 12 pediatric case-patients with Crimean-Congo hemorrhagic fever, Turkey*

Case no.	Age, y/sex	Initial signs and symptoms	Physical findings	FFPT/PT	Ribavirin duration, d	IVIG duration, d	Steroids duration, d	Complications
1	15/M	Fever, myalgia, headache	HSM, petechia	+/+	9	5	8	Transient bradycardia
2	7/F	Fever, malaise, nausea	HSM, petechia, somnolence	+/+	10	5	10	Transient adrenal insufficiency
3	12/M	Fever, headache, myalgia	HSM	+/−	7	1	3	None
4	8/M	Fever, malaise, nausea	HSM, petechia, confusion	+/+	2	4	10	Transient bradycardia
5	10/F	Fever, headache	HSM	+/−	5	1	10	None
6	13/M	Fever, headache	HSM, petechia	+/−	5	1	9	Transient bradycardia
7	15/M	Fever	HSM	+/−	5	1	5	Transient bradycardia
8	15/M	Fever, somnolence	HSM, petechia, confusion	+/−	4	1	8	Transient bradycardia
9	11/M	Fever, malaise	HSM, petechia	+/−	5	5	10	Transient bradycardia, transient adrenal insufficiency
10	15.5/M	Fever, headache, vomiting, diarrhea	HSM	+/−	5	1	5	Transient bradycardia
11	14/M	Fever, vomiting, diarrhea	HSM, petechia	+/−	5	1	5	Transient bradycardia
12	7/F	Fever, malaise	HSM, petechia	+/+	5	1	5	None

*All 12 case-patients survived. IVIG, intravenous immunoglobulin; FFPT, fresh frozen plasma transfusion; PT, platelet transfusion; HSM, hepatosplenomegaly; +, transfusion administered; –, transfusion not administered.

### Laboratory Findings 

All patients had leukopenia, lymphopenia, neutropenia, and thrombocytopenia when initially seeking treatment; 10/12 (83%) had normal hemoglobin levels. Other laboratory findings varied at admission and each time interval (Table 2). We observed that all patients deteriorated clinically over the course of the first time interval, consistent with their worsening laboratory tests. Except for triglycerides, fibrinogen, ESR, ALT, and AST, all laboratory parameters were at their worst levels during the first time interval; ALT and AST levels were at their highest during the second time interval, and ESR, fibrinogen, and triglycerides reached their maximum levels during the third interval. Increases were not statistically significant for ALT (p = 0.76), AST (p = 0.138), or fibrinogen (p = 0.07) but were for both ESR (p = 0.016) and triglycerides (p = 0.001). Over the third time interval, neutrophil (p = 0.134) and lymphocyte (p = 0.105) counts did not differ significantly, but both platelet (p = 0.045) and erythrocytes (p = 0.05) counts increased significantly. All patients had a significant decline in hemoglobin levels during the third time interval (p = 0.001). 

**Table 2:** Laboratory parameters for 12 pediatric case-patients with Crimean-Congo hemorrhagic fever at hospital admission and for 3 subsequent time intervals, Turkey*

Parameters, units	Reference range	Admission, median ±SD (min–max)	1st interval, median ±SD (min–max)	2nd interval, median ±SD (min–max)	3rd interval, median ±SD (min­–max)
Hemoglobin, g/dL	11.1–14	13.95 ±1.57 (11.30–16.60)	13.90 ±1.74 (10.70–16.20)	12.80 ±1.57 (9.40–15.30)	12.70 ±2.07 (8.50–15.80)
Leukocytes, × 10^3^ cells/μL	5–13	2.25 ±1.01 (1.10–4.70)	1.85 ±0.42 (1.30–2.70)	2.00 ±1.30 (1.10–4.10)	3.60 ±1.97 (2.60–7.60)
Neutrophils, × 10^3^ cells/μL	5–13	1.66 ±1.08 (0.25–3.89)	1.28 ±0.59 (0.20–1.87)	1.00 ±0.83 (0.33–2.96)	2.04 ±1.36 (0.58–4.29)
Lymphocytes, × 10^3^ cells/μL	1–5	0.46 ±0.26 (0.23–1.11)	0.46 ±0.25 (0.21–1.00)	0.64 ±0.50 (0.24 ±1.80)	0.83 ±0.49 (0.38–1.93)
Platelets, × 10^3^ μL	159–388	55.00 ±36.38 (13.00–144.00)	40.50 ±39.34 (11.00–127.00)	58.50 ±33.01 (22.00–116.00)	91.00 ±52.18 (33.00–219.00)
aPTT, s	22.5–32	34.70 ±7.28 (14.80–44.90)	33.10 ±6.92 (25.90–46.10)	25.55 ±3.33 (20.20–29.50)	21.90 ±4.90 (19.90–32.20)
INR	0.8–1.2	1.30 ±0.23 (0.96–1.70)	1.30 ±0.27 (0.96–1.89)	0.92 ±0.14 (0.85–1.37)	0.92 ±0.05 (0.72–1.00)
Fibrinogen, mg/dL	180–350	209.81 ±48.47 (153.68–323.81)	198.33 ±48.97 (140.84–303.00)	231.28 ±6,848.78 (170.91–22,691.00)	213.40 ±41.43 (153.29–286.21)
D-dimer, mg/dL	0–0.55	7.16 ±27.89 (1.48–67.15)	5.71 ±26.28 (1.89–78.48)	1.91 ±1.03 (0.79–4.45)	2.21 ±3.35 (0.56–10.68)
ALT, U/L	<39	65.00 ±159.188 (18.00–580.00)	58.50 ±174.94 (19.00–635.00)	129.00 ±86.74 (32.00–354.00)	143.00 ±79.78 (29.00–253.00)
AST, U/L	<51	162.50 ±443.42 (45.00–1648.00)	159.50 ±500.83 (47.00–1,818.00)	187.50 ±131.10 (72.00–519.00)	140.00 ±90.54 (52.00–359.00)
CK, U/L	<145	205.00 ±2,237.28 (69.00–7,297.00)	266.50 ±2,136.11 (54.00–6,292.00)	130.50 ±116.01 (60.00–364.00)	96.00 ±99.61 (42.00–301.00)
Triglyceride, mg/dL	<150	93.50 ±53.29 (43.00–192.00)	118.00 ±35.28 (46.00–142.00)	190.00 ±56.69 (130.00–304.00)	220.50 ±92.75 (91.00–378.00)
LDH, U/L	110–295	577 ±528.32 (288.00–2,091.00)	651.00 ±49.69 (295.00–1,757.00)	525.00 ±214.18 (313.00–1,089.00)	428.00 ±206.11 (212.00–848.00)
C-reactive protein, mg/dL	0–0.5	1.24 ±2.08 (0.10–6.95)	0.54 ±0.56 (0.12–1.85)	0.17 ±0.10 (0.03–0.39)	0.14 ±0.10 (0.02–0.4)
ESR, mm/h	0–20	3.00 ±5.54 (2.00–21.00)	8.00 ±7.79 (2.00–23.00)	22.00 ±12.74 (12.00–49.00)	36.00 ±15.40 (7.00–56.00)
Ferritin, μL	11–307	2,816.00 ±5,779.20 (309.00–21,690.00)	4,257.00 ±12,330.80 (648–40,455)	2,762.50 ±5,423.00 (558.00–18,772.00)	1,619.50 ±2,822.24 (404.00–9,981.00)
*First time interval, 68–72 h after baseline; second, 120–132 h; third, 156–180 h. aPTT, activated partial thromboplastin time; INR, international normalized ratio; ESR, erythrocyte sedimentation rate; AST, aspartate aminotransferase; ALT, alanine aminotransferase; CK, creatine kinase; LDH, lactate dehydrogenase.					

By the third time interval, INR was within normal range for all patients and aPTT levels for 91.7% (11/12), but only 8.3% (1/12) of patients had normal D-dimer levels. Declines in all 3 coagulation parameters over time were significant (p = 0.005 for INR, p = 0.002 for aPTT, and p = 0.001 for D-dimer). Median CRP levels were within normal limits for all 3 intervals, but change over time was significant (p = 0.001). Serum ferritin, CK, and LDH levels peaked during the first time interval and declined significantly over time (p = 0.035 for ferritin, p = 0.01 for CK, and p = 0.005 for LDH). Although nearly half (5/12) of patients had normal CK values at the third time interval, LDH and ferritin levels were above normal in all patients. We performed bone marrow aspiration on 10/12 (83%) patients, all of whom exhibited hemophagocytosis within 24–48 hours after admission. 

### Treatment

We administered 2 g/kg body weight intravenous immunoglobulin to all patients within 12 hours of admission, either as a 48-hour infusion or 400–500 mg/kg/day over 4–5 days. As part of HLH treatment for all patients, we administered dexamethasone to 8 patients and methylprednisolone to 4. Initial doses were 10 mg/m^2^ body surface area for dexamethasone and 2 mg/kg/day for methylprednisolone. Median ±SD initiation time for corticosteroid treatment was 25 ±3.86 hours (range 24–36 hours) and median duration of treatment was 8 ±2.57 days (range 3–10 days). We administered antiviral treatment (ribavirin) to all patients for a median of 5 ±2.54 days (range 2–10 days). We gave fresh frozen plasma and vitamin K to all patients; 3 (25%) required platelet transfusions. Nine (75%) patients experienced transient bradycardia during the treatment, and 2 (16.7%) had transient adrenal suppression. All 12 patients survived.

### Serum Cytokine/Chemokine Levels 

Cytokine/chemokine values varied both over time and between patient and control groups (Table 3). CCL5 levels were not evaluated further after they measured above upper limits indicated by the manufacturer in 6/11 control patients. 

**Table 3:** Median cytokine/chemokine levels of 9 pediatric case-patients with Crimean-Congo hemorrhagic fever and 11 healthy controls, Turkey*

Cytokine/ chemokine	1st time interval, pg/mL (range)	p value†	2nd time interval, pg/mL (range)	p value†	3rd time interval, pg/mL (range)	p value†	Controls, pg/mL (range)
IL-1β	31.19 (0–246.51)	0.94	76.97 (0–259.83)	**0.0014 **	50.10 (7.99–300.16)	0.5019	49.13 (0–743.64)
INF-α	105.88 (22.68–239.73)	**<0.0001 **	26.03 (8.95–94.97)	**0.0031 **	20.26 (99.08–26.45)	**0.0057 **	6.98 (2.43–25.41)
INF-γ	36.03 (16.94–106.43)	**0.0097 **	44.07 (9.59–157.74)	**0.0097 **	32.99 (13.91–286.60)	**0.0200 **	8.33 (1.89–39.78)
TNF-α	7.10 (0–29.84)	0.1747	31.51 (5.77–105.14)	0.6556	30.90 (14.22–290.76)	0.1519	10.74 (0–200.89)
MCCL2	1,947.00 (638.74–3,342.00)	**0.0125**	487.64 (247.30–4,974.00)	0.2947	396.94 (212.85–1,477.00)	0.0674	893.33 (477.73–1,101.00)
IL-6	115.30 (13.58–808.78)	0.0674	51.08 (13.19–717.75)	0.1308	39.87 (14.27–266.12)	0.1754	21.99 (2.92–1,297.00)
CXCL8	372.79 (35.34–4,712.00)	0.3312	130.62 (30.35–3,265.00)	0.6556	11.60 (61.16–3,619.00)	0.8238	116.79 (0–26,173.00)
IL-10	144.98 (16.73–1,194)	**0.0005 **	223.72 (161.65–1,355.00)	**<0.0001 **	146.44 (68.54–545.34)	**<0.0001 **	14.46 (0–40.00)
IL-12p70	5.74 (0–20.37)	0.8662	9.46 (3.59–86.07)	0.2299	11.42 (5.38–70.99)	0.1119	5.72 (0–18.11)
IL-17α	0.41 (0–4.59)	0.6406	2.05 (0.19–7.97)	0.4893	1.36 (0.16–395.36)	0.4893	0.67 (0–6.16)
IL-18	2,862 (857.42–4,179.00)	**0.0012 **	1,208 (55.17–3,818.00)	**0.0012 **	1,124.00 (623.86–6,374.00)	**0.0381 **	66.79 (299.29–1,779.00)
IL-23	10.66 (2.63–38.06)	0.5508	31.85 (6.05–43.74)	0.1302	23.51 (6.59–171.03)	0.1513	7.02 (0–54.01)
IL-33	33.06 (0–273.62)	0.6550	75.26 (29.76–496.32)	**0.0251 **	147.45 (36.87–500.62)	**0.0057 **	19.41 (0–161.00)
CXCL10	3,495.00 (1,677.00–5,475.00)	**<0.0001 **	1,674.00 (1,336.00–3,975.00)	**<0.0001 **	1,798.00 (972.37–2,620.00)	**<0.0001 **	215.35 (123.96–396.45)
CCL11	244.95 (81.83–352.51)	>0.9999	142.16 (90.23–237.45)	**0.0310 **	182.57 (72.10–260.51)	0.0952	219.07 (100.08–452.04)
CCL17	99.34 (62.75–175.50)	**<0.0001 **	101.56 (54.95–209.21)	**<0.0001 **	112.09 (26.97–258.12)	**<0.0001 **	766.77 (321.96–1,897.00)
RANTES	3,727.00 (2,357.00–4,642.00)	0.3455	2,849.00 (1,674.00–4,642.00)	**0.0139 **	2,045.00 (709.44–4,642.00)	**0.0048 **	161.00 (161.00)
MIP-1	95.90 (0–236.97)	0.7609	44.72 (0–2,605.00)	0.3269	124.29 (0–1,638.00)	0.1718	15.05 (0–2,112.00)
MIG	471.00 (296.89–791.29)	0.6027	369.51 (214.11–759.69)	0.7664	660.36 (292.87–851.42)	0.1119	382.81 (249.06–3,035.00)
CXCL5	203.91 (59.04–471.44)	**<0.0001 **	119.60 (35.72–441.08)	**<0.0001 **	103.63 (19.16–591.52)	**<0.0001 **	1,209.00 (467.16–1,875.00)
MIP-3α	130.05 (2.55–231.59)	**0.0310 **	80.30 (18.10–396.55)	0.2014	46.17 (14.45–329.60)	0.2947	41.81 (4.87–156.68)
GRO-α	218.75 (53.5–481.01)	**0.0251 **	131.41 (65.77–437.74)	**0.0008 **	145.73 (65.98–441.94)	**0.0016 **	370.46 (260.79–805.93)
CXCL11	1,332.00 (506.05–6,571.00)	**<0.0001**	338.10 (127.82–926.57)	**<0.0001 **	315.48 (178.08–515.34)	**<0.0001 **	50.29 (24.06–124.47)
MIP-1β	50.36 (26.79–272.66)	0.7103	28.82 (13.30–300.44)	0.2610	26.87 (9.81–405.40)	0.2610	44.54 (13.26–406.35)

*First time interval, 68–72 h after baseline; second, 120–132 h; third, 156–180 h. Bold indicates significance. CCL, CC chemokine ligand; CXCL, C-X-C motif chemokine ligand; IL, interleukin; INF, interferon; TNF, tumor necrosis factor; MCP, monocyte chemoattractant protein; IP, IFN-γ-inducible protein; TARC, hymus- and activation-regulated chemokine; RANTES, regulated upon activation, normal t cell expressed and presumably secreted; MIP, macrophage inflammatory proteins; MIG, monokine-induced γ interferon; ENA, epithelial neutrophil-activating; GRO, growth related protein; I-TAC, interferon-inducible T-cell alpha chemoattractant.

#### First Time Interval, 68–72 Hours

From beginning to end of the first time interval, 11/23 cytokine/chemokine levels differed significantly. Significantly elevated cytokines/chemokines were those associated with innate and adaptive T-helper 1 immune response: IFN-α (p<0.001), IFN-γ (p = 0.001), CXCL10 (p<0.001), and CXCL11 (p<0.001); IL-18 (p = 0.001) and CCL20 (formerly MIP-3α; p = 0.031), which regulate monocyte migration and the activation of macrophages and natural killer cells; CCL2 (p = 0.013), which acts through T-helper 2 cells; and antiinflammatory cytokine IL-10 (p = 0.0005). Patient CXCL1 (p = 0.025), CXCL5 (p <0.001), and CCL17 (p<0.001) were significantly lower than those for controls (Table 3; Figure).

**Figure:**
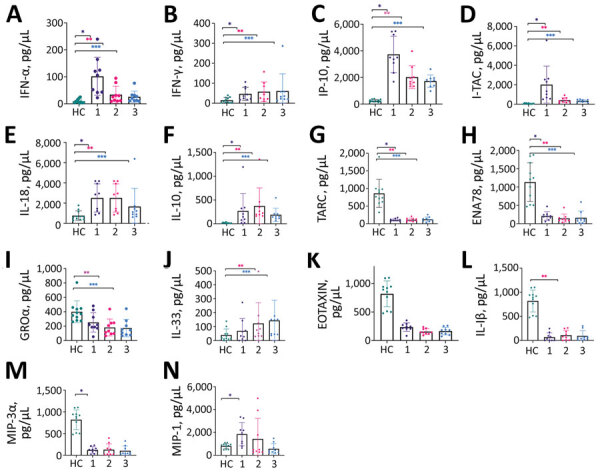
Quantitative levels of cytokines/chemokines among 12 case-patients with CCHF for 3 time intervals compared with results for controls, Turkey. 1, 1st time interval, 68–72 hours after admission (black); 2, 2nd time interval, 120–132 hours after admission (red); 3, 3rd time interval, 156–180 hours after admission (blue). Controls are shown in green. Time intervals contributing to significant changes designated by number of asterisks (*). *1st time interval; **2nd time interval; ***3rd time interval. CCL, CC chemokine ligand; CXCL, C-X-C motif chemokine ligand; ENA, epithelial neutrophil-activating; HC, healthy controls; IL, interleukin; IFN, interferon; IP, IFN-γ-inducible protein; I-TAC, interferon-inducible T-cell alpha chemoattractant; RANTES, regulated upon activation, normal t cell expressed and presumably secreted; MIP, macrophage inflammatory proteins; NA, not applicable; TARC, hymus- and activation-regulated chemokine.

#### Second Time Interval, 120–132 Hours

From beginning to end of the second time interval, 12/23 cytokines/chemokines differed significantly. IL-1β (p = 0.001) and IL-33 (p = 0.025), which activate T-helper 2 response, were elevated. In addition, IFN-α (p = 0.003), IFN-γ (p = 0.009), CXCL10 (p <0.001), CXCL11 (p <0.001), IL-18 (p = 0.001), and IL-10 (p <0.001) remained significantly elevated. CCL11, which is chemotactic for eosinophils, together with CXCL5 (p = 0.031), CCL17 (p <0.001), and CXCL1, were significantly lower compared with the controls (Table 3; Figure). 

#### Third Time Interval, 156–180 Hours

From beginning to end of the third time interval, except for IL-1β (p = 0.502) and CCL11 (p = 0.095), the same cytokines/chemokines were elevated as in the second time interval: IFN-α (p = 0.006), IFN-γ (p = 0.020), CXCL10 (p <0.001), CXCL11 (p <0.001), IL-18 (p = 0.038), IL-10 (p <0.001) and IL-33 (p = 0.006). Moreover, the same cytokines/chemokines remained lower than among the control group as in the second time interval: CXCL5 (p <0.001), CCL17 (p <0.001), and CXCL1 (p = 0.002) (Table 3; Figure). 

### Serum Cytokine/Chemokine Differences Based on Time Intervals

Five cytokines/chemokines, IFN-α (p = 0.016), CXCL10 (p = 0.016), CXCL11 (p = 0.016), CCL11 (p = 0.016), and CCL2 (p = 0.003), differed significantly depending on the time interval in which they were measured. IFN-α, CXCL10, and CXCL11, were significantly high throughout all 3 intervals, although highest during the first time interval. In contrast, CCL2 was significantly elevated only during the first time interval (Table 3). 

### Serum Cytokine/Chemokine Correlation with Laboratory Parameters

Some cytokines/chemokines were correlated with CCHF patient laboratory parameters depending on time interval (Table 4). Fibrinogen correlated with IL-18 during the first time interval, also with INF-α, IFN-γ, and IL-33 during the second interval. IL-18 and IL-33 were the only cytokines that correlated with ALT. Platelets correlated with CCL17 during the first and second time intervals, whereas they correlated with IL-10 during the third. CCL17 was the only chemokine that correlated with AST. Triglycerides correlated with INF-α, IFN-γ, IL-33, IL-1β, CXCL10, and CXCL11. LDH correlated with CCL3, IL-1β, CXCL11, CCL17, and CCL11. Finally, neutrophils correlated with CCL3 and IL-18, and ferritin with CCL3, IFN-γ, and CCL11. CXCL5 was the only chemokine that correlated with hemoglobin and ESR levels. 

**Table 4:** Variations in correlation of laboratory parameters with cytokines/chemokines for 12 pediatric case-patients with Crimean-Congo hemorrhagic fever, based on time interval, Turkey*†

Cytokine/chemokine	Neutrophil count	Platelet count	Fibrinogen	D-dimer	a-PTT	INR	Ferritin	LDH	ALT	TG
IFN-α	NA	NA	r2 = 0.726; p2 = 0.027	NA	NA	r1 = 0.720; p1 = 0.029	NA	NA	NA	r2 = 0.840; p2 = 0.018
IFN-γ	NA	NA	r2 = 0.770; p2 = 0.015	NA	NA	NA	r1 = 0.0857; p1 = 0.007	NA	NA	r2 = 0.863; p2 = 0.012
CXCL10	NA	NA	NA	r3 = 0.669; p3 = 0.049	r2 = 0.677; p2 = 0.045	NA	NA	NA	NA	r1 = −0.920; p1 = 0.009
CXCL11	NA	NA	NA	NA	NA	NA	NA	r3 = −0.716; p3 = 0.046	NA	r1 = −0.851; p1 = 0.032
MIP-3α	r1 = −0.783; p1 = 0.013	NA	NA	NA	NA	NA	r1 = 0.771; p1 = 0.025	r1 = 0.667; p1 = 0.05	NA	NA
IL-1β	NA	NA	NA	NA	NA	NA	NA	NA	NA	r2 = 0.883; p2 = 0.009
lL-10	NA	r3 = −0.748; p3 = 0.021	NA	NA	NA	NA	NA	NA	NA	r2 = 0.870; p2 = 0.011
IL-18	r1 = −0.721; p1 = 0.029	r1 = −0.762; p1 = 0.017;	r1 = −0.676; p1 = 0.046; r2 = 0.693; p2 = 0.038	NA	NA	NA	NA	r1 = 0.670; p1 = 0.048	r2 = 0.688; p2 = 0.049	NA
IL-33	NA	NA	r2 = 0.943; p2 = 0.001	NA	NA	NA	NA	NA	r2 = 0.739; p2 = 0.023	r2 = 0.851; p2 = 0.015
CCL17	NA	r1 = 0.823; p1 = 0.006; r2 = 0.851; p2 = 0.004	NA	NA	r1 = −0.733; p1 = 0.025	NA	NA	r3 = −0.907; p3 = 0.002	NA	NA
CCL11	NA	NA	NA	r2 = −0.758; p2 = 0.018; r3 = −0.668; p3 = 0.049	NA	NA	r2 = −0.681; p2 = 0.043	r2 = −0.669; p2 = 0.049; r3 = −0.895; p3 = 0.003	NA	NA
ENA78	NA	NA	NA	NA	NA	NA	NA	NA	NA	NA

*First time interval, 68–72 h after baseline; second, 120–132 h; third, 156–180 h. CCL, CC chemokine ligand; CXCL, C-X-C motif chemokine ligand; ENA, epithelial neutrophil-activating; IL, interleukin; IFN, interferon; IP, IFN-γ-inducible protein; I-TAC, interferon-inducible T-cell alpha chemoattractant; RANTES, regulated upon activation, normal t cell expressed and presumably secreted; MIP, macrophage inflammatory proteins; NA, not applicable; TARC, hymus- and activation-regulated chemokine. †r1, correlation coefficient during the first time interval; p1, asymptotic significance (2-tailed) during the first time interval; r2, correlation coefficient during the second time interval; p2, asymptotic significance (2-tailed) during the second time interval; r3, correlation coefficient during the third time interval: p3, asymptotic significance (2-tailed) during the third time interval.

## Discussion

Since CCHF was first identified in Turkey, children have been observed to have milder disease (*19**,**20*). During the past 2 decades, studies have shown that the course and outcome of CCHF depend on viral load, host genetic factors, and host immune response, together with the level of release of cytokines/chemokines (*10**,**12**,**14**,**21*). Furthermore, the association of hemorrhagic fevers with HLH suggests the involvement of cytokines/chemokines in the pathogenesis of the disease (*22**–**25*). One study reported HLH in 50% of adult CCHF patients (*26*); however, a case report describing an adult CCHF patient with HLH reported HLH as a rare condition (*27*). HLH is rarely reported among pediatric patients (*28*). 

After the official announcement of the COVID-19 pandemic in Turkey on March 11, 2020, we began to observe certain changes in pediatric CCHF cases. The first noticeable change was admission of patients earlier in the year. Although ticks are known to emerge as early as March, the CCHF season in Turkey runs May–September, with June and July as the peak months (*26**,**29**–**31*). During the study period, the first 2 cases were admitted in April, earlier than expected, probably because the patients had relocated to endemic rural valley districts as a result of restrictions and early school closures because of COVID-19. 

A second change was observed in clinical manifestations. All 12 CCHF case-patients in the study manifested >1 severe disease sign fulfilling the criteria for HLH: all had fever and hepatosplenomegaly, headache was the next most common complaint, most patients had petechiae, and 3 had neurologic findings at admission; none had tonsillopharyngitis. In previously published research on pediatric CCHF, the most common symptoms reported were fever and nausea, and tonsillopharyngitis was observed in a substantial percentage of patients, but hepatomegaly and splenomegaly were rarely reported (*20**,**32*). 

Cytokines/chemokines play substantial roles in the pathogeny of viral hemorrhagic fevers, and previous work has proposed that CCHF cytokine profiles are similar to cytokine profiles of other hemorrhagic fevers (*13**,**33*). Studies on the cytokine and chemokine profiles of CCHF patients have mainly been conducted in adults, and the levels of the inflammatory cytokines IL-6 and CXCL8 have been proposed as correlated with severe and fatal CCHF (*13*). Contrary to those findings, in our study, neither IL-6 nor CXCL8 was elevated in any patient during any time interval. Our finding that IL-6 and TNF-α levels were not elevated was consistent with a previous pediatric study (*15*). However, the finding on IL-6 levels differed from another pediatric study in which pediatric CCHF patients were reported to have elevated levels of IL-6 and IL-10 compared with control groups (*34*). The difference might have been because of timing and a single measurement of cytokines/chemokines. In our study, IFN-α, IFN-γ, CXCL10, and CXCL11 were elevated during all 3 time intervals, indicating the consequential role of the T-helper 1–dependent pathway in pediatric CCHF cases. 

Certain strains of CCHF virus have been shown in vitro to delay IFN production (*35*) by suppressing IFN-β-promoter–mediated gene expression, hampering the interferon type 1 response (*36*). However, that pattern seems not to have been the case with the pediatric CCHF patients in our study, among whom the IFN-γ pathway was consistently active. IFN-γ induces production of CXCL10 and CXCL11, structurally associated chemokines that attract C-X-C chemokine receptor type 3^+^ (CXCR3^+^) lymphocytes expressed by T-helper 1 cells (*37*). Our findings suggest that CXCL10 and CXCL11 also modulate T-helper 1–adaptive immunity for the progression of CCHF. IL-18, a proinflammatory cytokine that was elevated during all 3 time intervals, induced IFN-γ production by CD4 and CD8 T-cells and natural killer cells. IL-33, another inducer of IFN-γ production, was elevated in the second and third time intervals. IL-17, which acts in concert with TNF-α and IL-1β and is inhibited by INF-γ, was not elevated in any time interval. The levels of CXCL5, a chemokine inhibited by INF-γ, were significantly low in all time intervals, further supporting INF-γ upregulation. 

CCL2, which is expressed by macrophages in response to INF-γ, IL-6, TNF-α, IL-1β, and lipopolysaccharides, is another chemokine reported to correlate with the severity of CCHF (*12**,**13*). One study proposed that the course of disease in fatal cases was responsible for significantly high chemokines, mainly CCL2 in adult patients but not in pediatric patients, including patients with severe disease (*38*). This finding differs from our observation that all patients had high CCL2 levels during the first time interval; however, we did not observe elevated CCL2 levels in the subsequent intervals. MCP-1 binds to CCR2 and CCR5 receptors. Downregulation of CCR5 is known to be protective against HIV (*39*) and dengue virus (*40*); it has been reported that the lower the expression of CCR5, the better the outcome. Furthermore, proinflammatory cytokine CXCL1 acting through CCR2 levels was lower among patients than controls, suggesting that CCR2 receptors are downregulated in pediatric CCHF patients. 

Correlations between cytokine/chemokine levels and laboratory parameters varied. Although all patients fulfilled the HLH criteria, triglyceride levels were invariably low during the first time interval. In that period, triglyceride levels negatively correlated with CXCL10 and CXCL11, both chemokines known to cause hypertriglyceridemia. Another notable laboratory parameter was CRP, which did not correlate with any cytokines/chemokines. A recent report described CCHF patients misdiagnosed with MIS-C during the pandemic (*17*). Indeed, 3 case-patients in our study were referred with a preliminary diagnosis of MIS-C. However, CRP levels differed uniquely between CCHF and MIS-C cases. CRP levels of almost all the CCHF cases were within reference limits throughout all time intervals, but median CRP levels in MIS-C cases were low, at 18.7 mg/dL in 1 study (*41*) and 19.6 mg/dL in another (*42*). Meanwhile, the CRP levels of the MIS-C cases associated with HLH were 7.5–21.9 mg/dL (*43*). Combined with a tick-bite history, low CRP levels should raise suspicion of CCHF in pediatric patients with HLH in endemic regions. 

Among limitations of our study, the small number of CCHF patients, all of whom had severe disease, did not allow us to generalize findings to all CCHF patients. However, we believe that serial measurement of cytokines/chemokines in our study represents a notable advantage over studies that used a single measurement. Another limitation was that, although all patients had a cycle threshold value <20, it was not possible to have viral loads measured or CCHF virus strains sequenced because of increased laboratory workload during the COVID-19 pandemic.

In conclusion, our results show that children can have severe CCHF that manifests with HLH-like signs and symptoms. We observed strong IFN pathway activation and a T-helper 1–biased immune response. Downregulation of cytokines acting through CCR2 and CCR5 had a favorable effect among case-patients despite severe disease. In the future, larger controlled studies including CCHF pediatric patients with various disease severity should be conducted to verify the actual roles of different cytokine/chemokine profiles among children. Clinicians should be aware that children can manifest severe CCHF and that, in the context of the ongoing COVID-19 pandemic, those cases might be initially considered to be MIS-C. Our results further suggest that HLH secondary to CCHF can be successfully treated with intravenous immunoglobulin and steroid therapy. 
